# Association of Albumin‐To‐Creatinine Ratio With Diabetic Retinopathy Among US Adults (NHANES 2009–2016)

**DOI:** 10.1002/edm2.70029

**Published:** 2025-01-18

**Authors:** Han Dai, Ling Liu, Weiwei Xu

**Affiliations:** ^1^ Department of Endocrinology and Metabolism Second Affiliated Hospital of Chongqing Medical University Chongqing China; ^2^ Department of Ophthalmology Chongqing University Central Hospital, Chongqing Emergency Medical Center Chongqing China

**Keywords:** albumin‐to‐creatinine ratio, diabetes mellitus, diabetic retinopathy, glycated haemoglobin, inflammation, NHANES

## Abstract

**Objective:**

This study investigates the relationship between the albumin‐to‐creatinine ratio and diabetic retinopathy (DR) in US adults using NHANES data from 2009 to 2016. This study assesses the predictive efficacy of the urinary serum albumin‐to‐creatinine ratio (UACR/SACR Ratio) against traditional biomarkers such as the serum albumin‐to‐creatinine ratio (SACR) and urinary albumin‐to‐creatinine ratio (UACR) for evaluating DR risk. Additionally, the study explores the potential of these biomarkers, both individually and in combination with HbA1c, for early detection and risk stratification of DR.

**Methods:**

This cross‐sectional study analysed data from 2594 diabetic adults in the National Health and Nutrition Examination Survey (NHANES 2009–2016). Multivariate logistic regression models, adjusted for demographic (sex, age, race and education) and clinical factors (WBC, PLT, RDW, HbA1c, HBP and CHD), examined the associations between biomarkers and DR. Biomarkers were analysed both continuously and in quartiles to assess dose–response relationships. Receiver operating characteristic (ROC) curve analysis evaluated the predictive accuracy of individual biomarkers and combined models.

**Results:**

Elevated SACR levels were inversely related to DR risk, while UACR showed a positive correlation. The UACR/SACR ratio demonstrated superior predictive capability for DR compared to SACR and UACR alone. The most accurate predictive model combined SACR, UACR, UACR/SACR ratio and HbA1c (AUC = 0.614), highlighting DR development complexity. Subgroup analyses revealed stronger associations in participants aged < 60 years and those with hypertension (both *p* < 0.05), with more pronounced effects observed in males and Mexican Americans, while lifestyle factors showed no significant modifying effect.

**Conclusion:**

The UACR/SACR Ratio emerged as a potentially superior DR predictor, particularly in younger patients and those with hypertension, suggesting its utility in enhancing early detection and risk stratification. Comprehensive evaluation of renal function and glycaemic control biomarkers, considering age‐ and comorbidity‐specific patterns, could improve DR risk prediction and management. Future longitudinal studies should validate these findings, particularly in identified high‐risk subgroups, and investigate underlying mechanisms, potentially advancing personalised DR prediction and management strategies.

## Graphical abstract

This study investigates the relationship between albumin‐to‐creatinine ratio and diabetic retinopathy (DR) in US adults using NHANES data from 2009 to 2016. It assesses the predictive efficacy of the urinary serum albumin‐to‐creatinine ratio (UACR/SACR Ratio) against traditional biomarkers for evaluating DR risk. The study finds that the UACR/SACR ratio could be a superior predictor for DR, suggesting its utility in enhancing early detection and risk stratification.

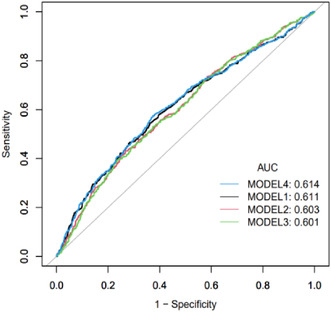

## 1 Introduction

Diabetic retinopathy (DR) is a common microvascular complication in people with diabetes, increasingly contributing to adult vision loss and blindness worldwide [1, 2]. Type 2 diabetes mellitus (T2DM) is characterised by a chronic inflammatory burden, which plays a crucial role in the development of its complications [3]. This inflammatory state contributes significantly to both microvascular and macrovascular complications [4].

DR development is closely associated with an increased inflammatory burden, as evidenced by elevated levels of inflammatory markers and cytokines in affected patients [5]. The inflammatory process contributes to retinal microvascular damage through multiple pathways, including endothelial dysfunction, basement membrane thickening and pericyte loss [6].

Furthermore, impaired renal function is strongly associated with the onset and progression of DR, and may serve as a predictor for its development [7, 8, 9, 10]. The urinary albumin‐to‐creatinine ratio (UACR), an indicator of renal function, is frequently elevated alongside endothelial dysfunction, potentially affecting the microvascular health of the kidneys and retina [11, 12, 13, 14]. Importantly, UACR has been linked with systemic inflammation [15], suggesting that inflammation may serve as a common pathway connecting albuminuria and DR development.

The complex interplay between systemic inflammation, renal dysfunction, and retinal damage provides a strong rationale for investigating the relationship between UACR and DR [16]. Understanding these connections could lead to more effective strategies for early detection and intervention in DR development. This study presents the urinary serum albumin‐to‐creatinine ratio (UACR/SACR ratio) as a new composite biomarker aimed at providing a comprehensive assessment of microvascular health in diabetic patients. By examining both individual biomarkers—SACR and UACR—and their combined ratio, our goal is to enhance the comprehensiveness of microvascular health assessment in diabetes.

Ultimately, this study seeks to advance the field of DR research by providing novel insights into the predictive utility of SACR, UACR and their ratios, both independently and in combination with HbA1c. Our findings may significantly impact DR prevention, early detection and treatment, leading to more effective and targeted healthcare interventions.

## 2 Materials and Methods

### 2.1 Study Design and Participant Recruitment

**FIGURE 1:**
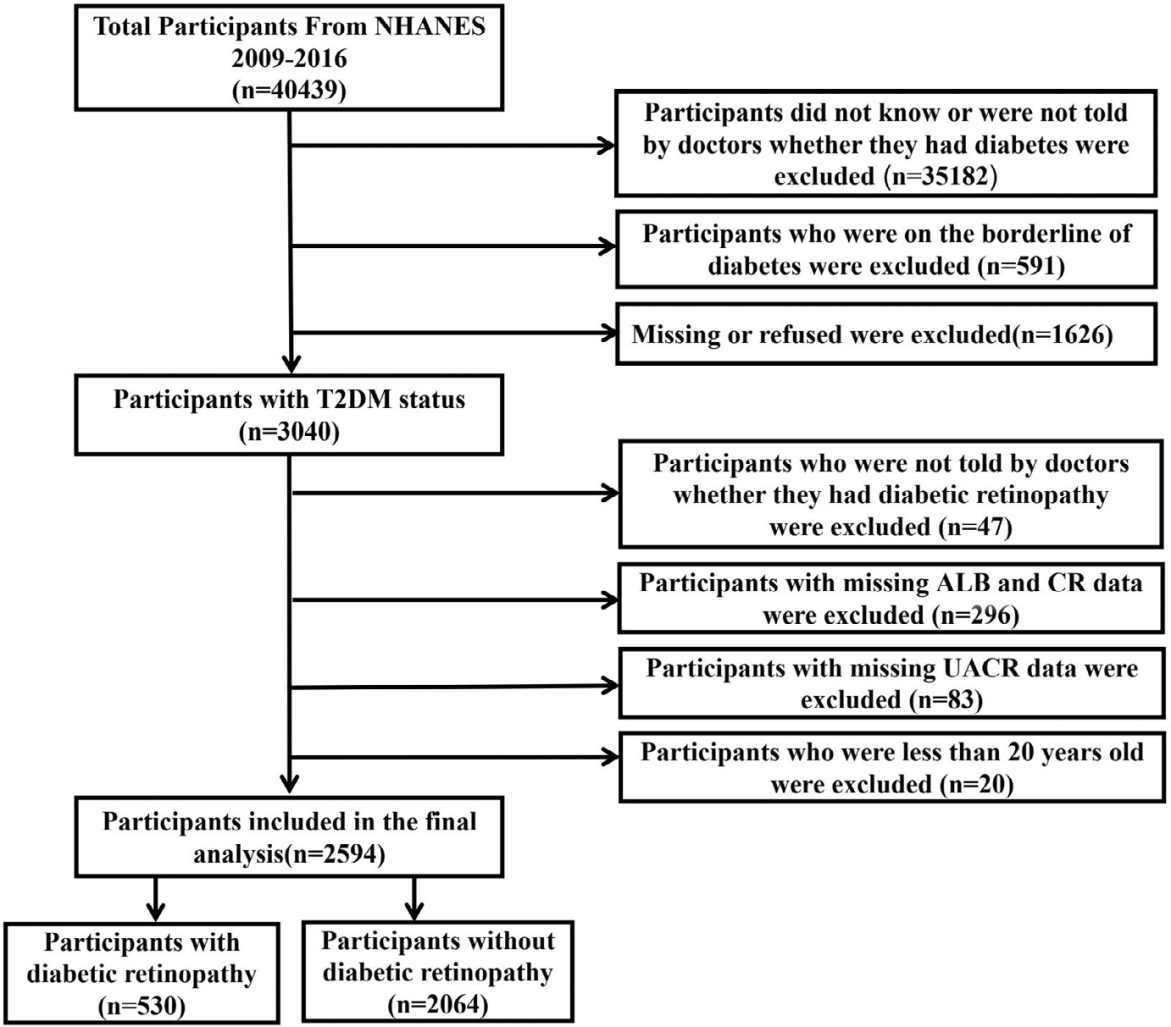
Illustration of the selection process flow chart. ALB, albumin; CR, creatinine; DM, diabetes mellitus; NHANES, National Health and Nutrition Examination Survey; UACR, urinary albumin‐to‐creatinine ratio.

This study utilised data from the National Health and Nutrition Examination Survey (NHANES) spanning 2009–2016. Our study initially included 3040 individuals who qualified as having diabetes mellitus (DM). According to the flow chart in Figure 1, participants were excluded if they had incomplete data on albumin (ALB), creatinine (CR) and UACR were under 20 years old or lacked medical confirmation of diabetic retinopathy (DR). The Institutional Review Board of the National Center for Health Statistics approved the study protocol, which complied with the Declaration of Helsinki. All participants provided informed consent before joining the study. Post‐participant selection, we focused on measuring critical biomarkers to evaluate their correlation with DR.

### 2.2 Exposure

Random urine samples were collected during the first visit to the mobile examination center (MEC) to assess urinary albumin and creatinine levels. These samples were frozen at −30°C for preservation and subsequently transported to the University of Minnesota in Minneapolis. Urinary albumin was measured via a solid‐phase fluorescence immunoassay, while creatinine was determined using a modified Jaffe kinetic method. The UACR was calculated by dividing urinary albumin concentration by urinary creatinine concentration.

Blood samples for serum albumin and creatinine analysis were collected during physical examinations at the MEC. Samples were collected in tubes containing ethylene diamine tetraacetic acid (EDTA) to prevent coagulation. Following processing and centrifugation to isolate the serum, the samples were frozen at approximately −70°C before being dispatched to the designated lab for further analysis. The DxC800 modular chemistry analyser, which employs the IDMS‐standardised Jaffe rate method, was used to quantify serum creatinine levels. The concentration of serum albumin was determined using the bichromatic digital endpoint method with the DxC800, a process that involves the interaction of albumin with Bromcresol Purple (BCP) dye. The ratio of serum albumin to creatinine was then computed by dividing the serum albumin level by the serum creatinine level.

The UACR/SACR ratio is calculated by dividing the UACR by the SACR.

### 2.3 DM and DR Definitions

Data from the National Health and Nutrition Examination Survey (NHANES) between 2009 and 2016 were used to identify individuals diagnosed with diabetes mellitus (DM) and diabetic retinopathy (DR) based on healthcare provider questionnaires.

### 2.4 Covariates

In this study, we accounted for various covariates including sex, age, racial background, body mass index (BMI), waist circumference (WAIST), tobacco use, alcohol consumption and educational attainment. Educational levels were categorised into four groups: primary school, secondary school, college level or higher and unrecorded. Racial categories included Mexican American, non‐Hispanic White, other Hispanic, non‐Hispanic Black and additional racial groups.

Tobacco use was evaluated via a questionnaire on cigarette smoking, asking participants about their lifetime consumption to distinguish never smokers from former and current smokers, using a threshold of 100 cigarettes. Former smokers were defined as those who had smoked at least 100 cigarettes but had ceased smoking, while current smokers were those who continued to smoke beyond the 100‐cigarette mark.

Alcohol consumption was evaluated via the ‘alcohol use’ questionnaire, which inquired about the frequency and average quantity of alcohol intake over the past 12 months. This information was used to estimate the weekly average of alcoholic drinks consumed, categorising participants into four groups: non‐drinkers (serving as the reference group), light drinkers, moderate drinkers and heavy drinkers. A standard drink was defined as 12 oz of beer, 5 oz of wine or 1.5 oz of spirits.

Laboratory measurements encompassed total cholesterol(TCH), triglycerides(TG), uric acid, white blood cell count (WBC), lymphocyte count (LYM), neutrophil count(NEN), platelet count (PLT), red cell distribution width(RDW), glycated haemoglobin(HbA1c), albumin(ALB), serum creatinine(SCR) and low‐density lipoprotein cholesterol (LDL), all analysed using standardised methods. We included chronic disease variables, hypertension (HBP) and coronary atherosclerotic heart disease (CHD), to examine their potential impact on the relationship between SACR, UACR, UACR/SACR ratio and DR.

### 2.5 Handling Missing Data

In the analysis of continuous variables such as age, BMI, WBC, LYM, NEN, RDW, PLT, TCH and HbAlc, which had a small number of missing values (less than 100), we employed mean imputation. For continuous variables with a higher number of missing data points (more than 100), namely PIR, WAIST, TG and LDL, we converted them into categorical variables. We addressed missing data by categorising it separately and applied the dummy variable method for imputation. Categorical variables such as drinking, smoking, HBP and CHD, with missing values, were assigned to a separate category, and the dummy variable method was used for interpolation.

### 2.6 Statistical Analysis

All analyses incorporated NHANES complex sampling design features, including sample weights, stratification and clustering, utilising the 2‐year MEC examination weights (WTMEC2YR) to ensure nationally representative estimates. Descriptive statistics were presented as weighted means ± standard deviations for continuous variables and weighted frequencies with percentages for categorical variables. Between‐group comparisons employed weighted t‐tests for continuous variables and weighted chi‐squared tests for categorical variables.

The associations between biomarkers (SACR, UACR and UACR/SACR Ratio) and DR were examined through multiple analytical approaches. Multivariate logistic regression analyses were conducted using three sequential models: Model 1 (unadjusted), Model 2 (adjusted for demographic factors: sex, age, race, education and PIR) and Model 3 (further adjusted for clinical parameters: WBC, PLT, RDW, HbA1c, HBP and CHD). Dose–response relationships were evaluated by categorising biomarkers into quartiles and conducting trend analyses.

Subgroup analyses assessed effect modification by key demographic and clinical characteristics, including age (< 60 vs. ≥ 60 years), sex, race/ethnicity, hypertension status and lifestyle factors. Effect modification was evaluated through multiplicative interaction terms in the fully adjusted model, with likelihood ratio tests comparing models with and without interaction terms. Forest plots were generated to visualise subgroup‐specific associations.

Non‐linear relationships between biomarkers and DR were explored using smoothing curve fitting. The predictive performance was assessed through receiver operating characteristic (ROC) curves, both for individual biomarkers and combined models incorporating multiple biomarkers with HbA1c. All analyses were performed using R version 4.2.0 (The R Foundation) and EmpowerStats software (X&Y Solutions Inc.), with statistical significance defined as a two‐sided *p* < 0.05.

## 3 Results

### 3.1 Participants Characteristics

Table 1 presents the medical and demographic features of 2594 individuals. Statistically significant differences were not observed in variables such as sex, age, BMI, WAIST, NEN, RDW, TG, LDL, TCH, drinking habits, smoking status and HBP. However, individuals with DR exhibited higher levels of HbA1c, SCR, UACR and the UACR/SACR ratio, which were statistically significant (*p* < 0.05). The prevalence of DR was also associated with a higher likelihood of CHD (*p* < 0.05) and a decrease with increasing levels of education (*p* < 0.05). Furthermore, patients with DR showed significantly lower values in PIR, WBC, LYM, PLT and ALB (*p* < 0.05).

**TABLE 1:** Demographic and clinical characteristics of participants with and without DR.

Characteristic	Total (*n* = 2594)	NDR (*n* = 2064)	DR (*n* = 530)	*p*
Age (years)	59.617 ± 13.273	59.591 ± 13.313	59.740 ± 13.080	0.83
PIR	2.672 ± 1.599	2.707 ± 1.583	2.502 ± 1.658	0.02
BMI (kg/m^2^)	32.941 ± 7.434	32.955 ± 7.343	32.873 ± 7.855	0.83
WAIST (cm)	111.253 ± 16.058	111.311 ± 15.864	110. 965 ± 16.982	0.69
WBC (10^3^/μL)	7.770 ± 2.264	7.824 ± 2.267	7.519 ± 2.235	< 0.01
LYM (10^3^/μL)	2.142 ± 0.978	2.168 ± 1.015	2.020 ± 0.770	< 0.01
NEN (10^3^/μL)	4.772 ± 1.757	4.796 ± 1.748	4.658 ± 1.795	0.13
PLT (10^3^/μL)	233.067 ± 70.389	234.331 ± 70.133	227.146 ± 71.278	0.05
RDW (%)	13.662 ± 1.349	13.653 ± 1.354	13.705 ± 1.325	0.45
TG (mmol/L)	1.809 ± 1.911	1.824 ± 1.993	1.730 ± 1.416	0.52
LDL (mmol/L)	2.579 ± 0.897	2.587 ± 0.876	2.538 ± 0.997	0.49
TCH (mmol/L)	4.645 ± 1.181	4.657 ± 1.176	4.588 ± 1.201	0.26
HbAlc (%)	7.379 ± 1.745	7.274 ± 1.709	7.872 ± 1.825	< 0.01
ALB (g/dL)	4.167 ± 0.342	4.185 ± 0.331	4.082 ± 0.380	< 0.01
SCR (mg/dL)	1.002 ± 0.614	0.960 ± 0.476	1.197 ± 1.019	< 0.01
SACR (g/mg)	4.167 ± 0.342	4.877 ± 1.480	4.389 ± 1.703	< 0.01
Uric acid (μmol/L)	340.529 ± 92.266	339.203 ± 90.420	346.759 ± 100.246	0.11
UACR (mg/g)	139.051 ± 650.288	108.773 ± 530.945	281.245 ± 1029.752	< 0.01
UACR/SACR ratio (mg^2^/g^2^)	81.129 ± 658.078	60.525 ± 611.626	177.889 ± 835.883	< 0.01
**Drinking habits (%)**				0.13
Light drink	59.235	58.477	63.764	
Moderate drink	28.877	29.870	22.948	
Heavy drink	11.888	11.654	13.288	
**Smoking status (%)**				0.60
Never smoker	75.64	75.19	77.77	
Former smoker	2.710	2.689	2.811	
Current smoker	21.654	22.122	19.420	
**Sex (%)**				0.41
Male	52.153	51.783	53.891	
Female	47.847	48.217	46.109	
**Race (%)**				0.01
Mexican American	10.142	10.021	10.711	
Other Hispanic	5.951	5.545	7.855	
Non‐Hispanic White	60.314	61.776	53.444	
Non‐Hispanic Black	14.654	14.257	16.515	
Other race	8.939	8.400	11.474	
**Education (%)**				< 0.01
Primary school	10.687	9.907	14.347	
Secondary school	35.952	34.648	42.075	
College or above	53.329	55.406	43.578	
Not recorded	0.032	0.039		
**HBP (%)**				0.54
Yes	68.166	67.736	70.182	
No	31.792	32.212	29.818	
Not recorded	0.042	0.051		
**CHD (%)**				0.02
Yes	10.865	10.088	14.515	
No	88.483	89.233	84.961	
Not recorded	0.652	0.680	0.524	
**Insurance (%)**				0.85
Yes	84.965	85.247	84.905	
No	15.035	14.753	15.095	
**Employment (%)**				0.06
Yes	35.586	39.375	34.780	
No	64.614	60.625	65.220	

*Note:* Mean ± SD for age, PIR, BMI, WAIST, WBC, LYM, NEN, PLT, RDW, TG, LDL, TCH, HbA1c, ALB, SCR, SACR, uric acid, UACR, UACR/SACR ratio. The *p* value, indicating statistical significance, was determined using a weighted linear regression model. For categorical variables such as sex, race, education, drinking habits, smoking status, HBP and CHD, the *p* values were ascertained through the application of a weighted chi‐squared test. We employed the following formulas: SACR (g/mg) is calculated by dividing serum albumin (g/dL) by serum creatinine (mg/dL). UACR (mg/g) = urinary albumin(mg/dL)/urine creatinine(g/dL). UACR/SACR ratio(mg^2^/g^2^) = UACR/SACR.

Abbreviations: ALB, albumin; BMI, body mass index; CHD, coronary atherosclerotic heart disease; HBAlc, glycated haemoglobin; HBP, hypertension; LDL, low‐density lipoprotein; LYM, lymphocytes; NEN, neutrophils; PIR, poverty income ratio; PLT, platelets; RDW, red cell distribution width; SACR, albumin‐to‐creatinine ratio; SCR, serum creatinine; TCH, total cholesterol; TG, triglycerides; UACR/SACR ratio, urinary serum albumin‐to‐creatinine ratio; UACR, urinary albumin‐to‐creatinine ratio; UA, uric acid; WAIST, waist circumference; WBC, white blood cell.

### 3.2 Association Between SACR and DR

**FIGURE 2:**
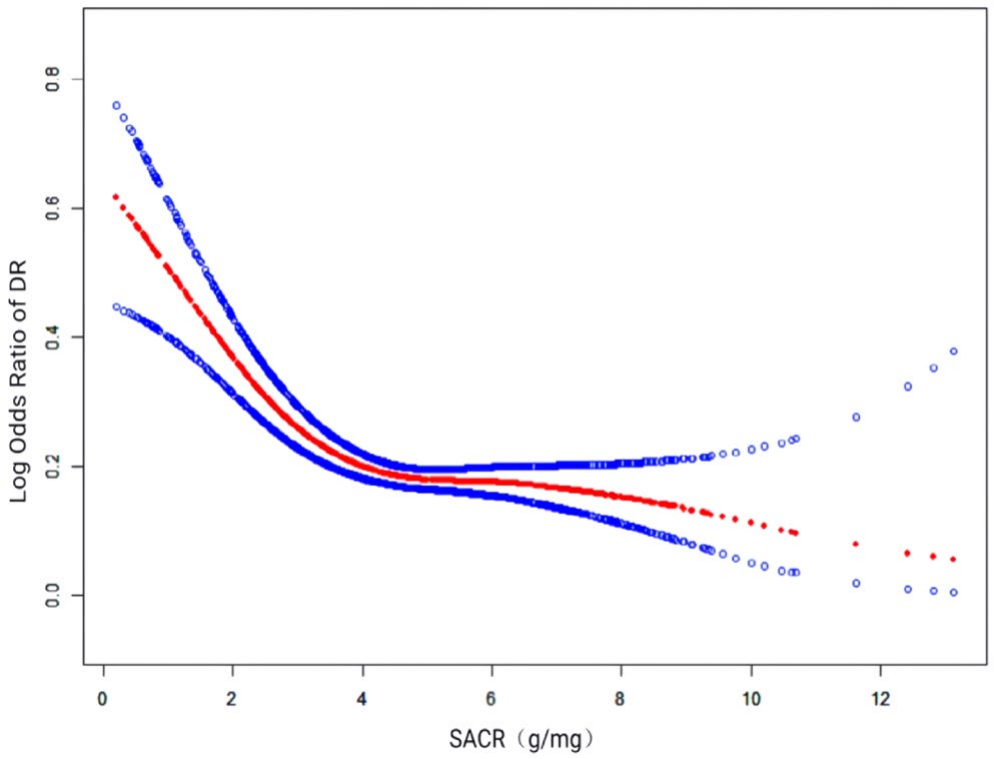
The nonlinear association between the SACR and DR. The solid red line represents the fitted smooth curve between the variables, with the blue areas indicating the 95% confidence intervals from the model fit. DR, diabetic retinopathy; SACR, albumin‐to‐creatinine ratio.

Our analysis utilising regression revealed a notably inverse correlation between the SACR and the likelihood of developing DR. In the non‐adjusted model (Model 1), the beta coefficient was 0.804 (95% CI: 0.736, 0.878), *p* < 0.001. In Model 2, after controlling for confounders including sex, age, race, education and PIR, the inverse association remained significant with a beta coefficient of 0.761 (95% CI: 0.696, 0.833), *p* < 0.001. Further adjustment for additional variables including WBC, PLT, RDW, HbAlc and history of HBP or CHD in the Model3 did not significantly alter the relationship, with a beta coefficient of 0.766 (95% CI: 0.698, 0.841), *p* < 0.001. Quartile stratification of SACR revealed a notable reduction in the odds ratio for DR as SACR levels increased across all models. The odds ratios for Q2, Q3, and Q4 were 0.390 (95% CI: 0.268–0.569), 0.503 (95% CI: 0.346–0.733) and 0.462 (95% CI: 0.317–0.675) respectively, all with *p* < 0.001, demonstrating a dose–response inverse relationship (Table 2). The smooth curve fitting analysis graphically confirmed the inverse association observed in the regression models. The curve showed a consistent decrease in DR risk as SACR levels increased, aligning with the quartile analysis findings (Figure 2).

**TABLE 2:** Correlation between the SACR and DR.

Exposure	Model 1			Model 2			Model 3		
	OR	95% CI	*p*	OR	95% CI	*p*	OR	95% CI	*p*
SACR	0.804	(0.736, 0.878)	< 0.001	0.761	(0.696, 0.833)	< 0.001	0.766	(0.698, 0.841)	< 0.001
**SACR quartile**									
Q1		Ref			Ref			Ref	
Q2	0.390	(0.268, 0.569)	< 0.001	0.368	(0.253, 0.534)	< 0.001	0.385	(0.265, 0.559)	< 0.001
Q3	0.503	(0.346, 0.733)	< 0.001	0.509	(0.349, 0.743)	< 0.001	0.547	(0.371, 0.807)	< 0.001
Q4	0.462	(0.317, 0.675)	< 0.001	0.403	(0.261, 0.621)	< 0.001	0.419	(0.269, 0.651)	< 0.001
*p* for trend		< 0.001			< 0.001			< 0.001	

*Note:* Model 1 unadjusted. Model 2 adjusted for sex, age, race, education and poverty income ratio. Model 3 was adjusted for variables including sex, age, race, education, poverty income ratio, white blood cell count, platelet count, red cell distribution width, glycated haemoglobin and a history of hypertension or coronary heart disease.

Abbreviations: CI, confidence interval; DR, diabetic retinopathy; OR, odds ratio; SACR, serum albumin‐to‐creatinine ratio.

### 3.3 Association Between UACR and DR

**FIGURE 3:**
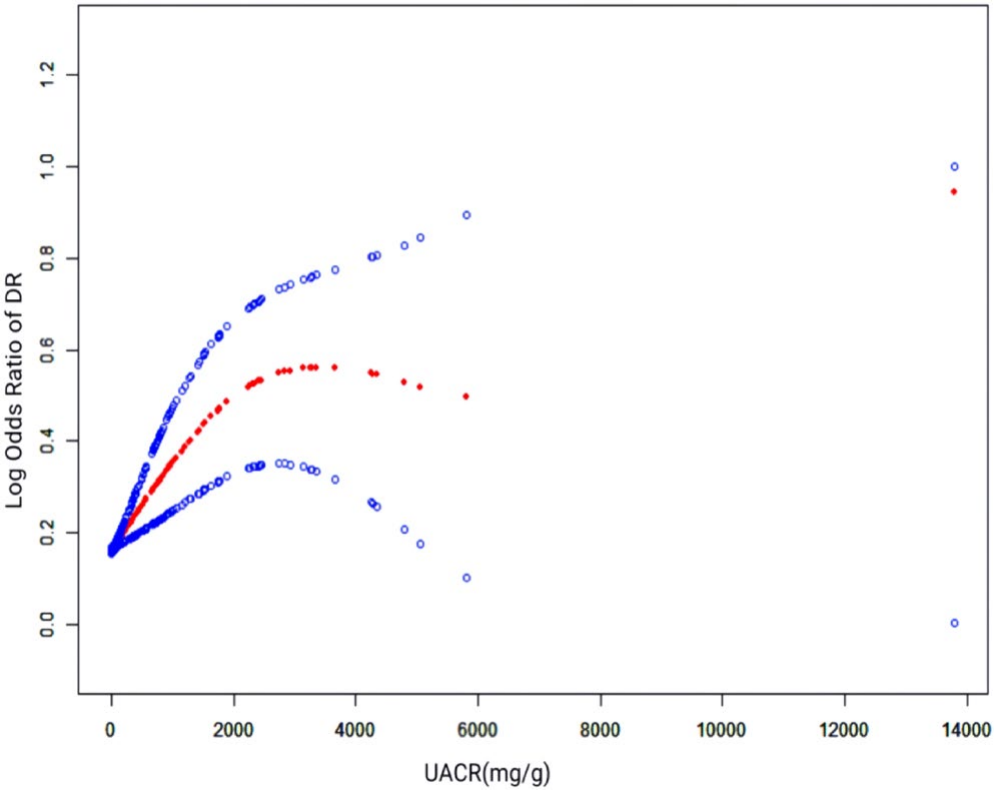
The nonlinear association between the UACR and DR. The solid red line represents the fitted smooth curve between the variables, with the blue areas indicating the 95% confidence intervals from the model fit. DR, diabetic retinopathy; UACR, urine albumin‐to‐creatinine ratio.

Regression analysis revealed a significant positive association between UACR and DR in all models. In Model 1 without adjustments, UACR was significantly associated with DR (OR = 1.000, 95% CI 1.000, 1.000, *p* = 0.003). Adjusting for demographic and clinical variables in Models 2 and 3 did not change the association, as UACR quartiles continued to show a progressive increase in ORs for DR, peaking in the highest quartile (Q4 OR = 2.272, 95% CI 1.624, 3.178, *p* < 0.001 for Model 3). The significant *p*‐value for trend across all models suggests a dose–response relationship (Table 3). Smooth curve fitting further validated the positive correlation between UACR and DR, showing an upward trend in DR risk with increasing UACR levels (Figure 3).

**TABLE 3:** Correlation between UACR and DR.

Exposure	Model 1			Model 2			Model 3		
	OR	95% CI	*p*	OR	95% CI	*p*	OR	95% CI	*p*
UACR	1.000	(1.000, 1.000)	0.003	1.000	(1.000, 1.000)	0.003	1.000	(1.000, 1.000)	0.006
**UACR quartile**									
Q1		Ref			Ref			Ref	
Q2	0.769	(0.517, 1.142)	0.019	0.719	(0.471, 1.099)	0.125	0.671	(0.439, 1.027)	0.065
Q3	1.620	(1.136, 2.311)	0.009	1.603	(1.144, 2.246)	0.007	1.442	(1.020, 2.038)	0.039
Q4	2.272	(1.624, 3.178)	< 0.001	2.213	(1.596, 3.070)	< 0.001	1.830	(1.284, 2.608)	0.001
*p* for trend		< 0.001			< 0.001			< 0.001	

*Note:* Model 1 unadjusted. Model 2 adjusted for sex, age, race, education and poverty income ratio. Model 3 was adjusted for variables including sex, age, race, education, poverty income ratio, white blood cell count, platelet count, red cell distribution width, glycated haemoglobin and a history of hypertension or coronary heart disease.

Abbreviations: CI, confidence interval; DR, diabetic retinopathy; OR, odds ratio; UACR, urinary albumin‐to‐creatinine ratio.

### 3.4 Association Between UACR/SACR Ratio and DR

**FIGURE 4:**
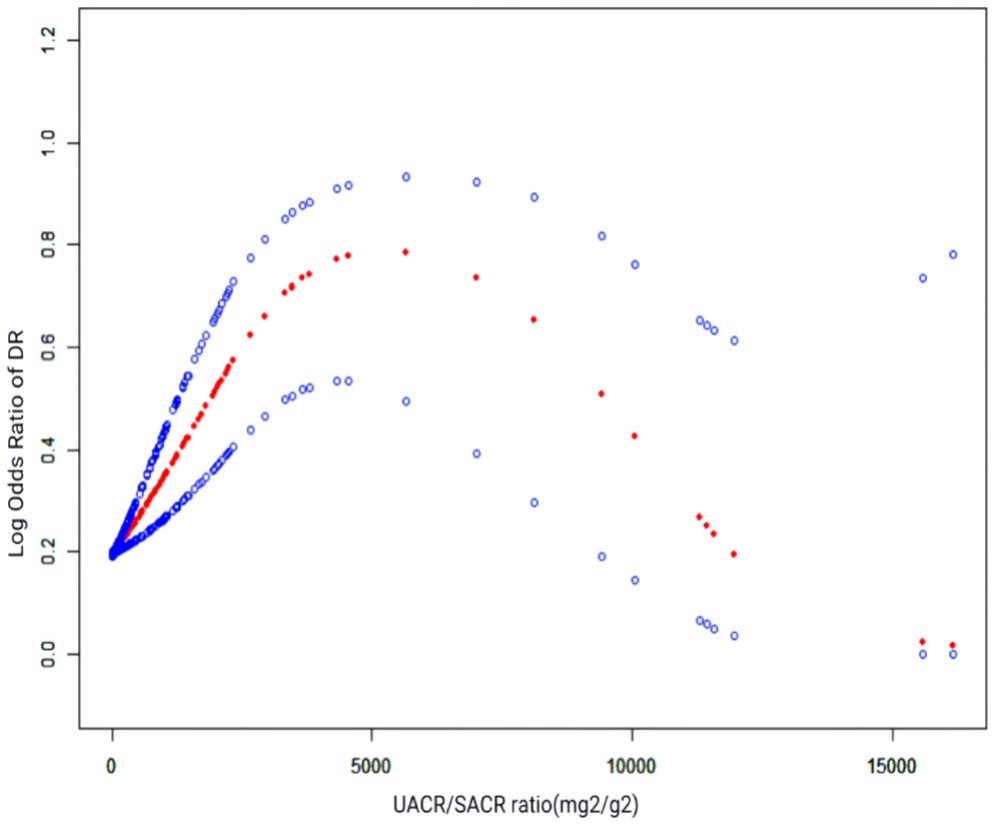
The nonlinear association between the UACR/SACR ratio and DR. The solid red line represents the fitted smooth curve between the variables, with the blue areas indicating the 95% confidence intervals from the model fit. DR, diabetic retinopathy; UACR/SACR Ratio, urine‐serum albumin‐to‐creatinine ratio.

Regression analysis showed a significant link between the UACR/SACR ratio and DR, with a notable risk increase in the higher quartiles. In Model 1 without adjustments, the UACR/SACR ratio was significantly associated with DR (OR = 1.000, 95% CI 1.000, 1.000, *p* = 0.043). This association persisted across all adjustment models, with the most pronounced increase in risk observed in the fourth quartile (Q4), where the ORs were 2.645, 2.766 and 2.382 for Models 1, 2 and 3, respectively, all with *p* < 0.001.The consistent *p* for trend across models (all *p* < 0.001) underscores a robust dose–response relationship (Table 4). The smooth curve fitting analysis visually confirmed a positive association between UACR/SACR ratio and DR. The curve indicated a progressive increase in DR risk with rising UACR/SACR ratio levels, aligning with the quartile analysis (Figure 4).

**TABLE 4:** Correlation between UACR/SACR ratio and DR.

Exposure	Model 1			Model 2			Model 3		
	OR	95% CI	*p*	OR	95% CI	*p*	OR	95% CI	*p*
UACR/SACR ratio	1.000	(1.000, 1.000)	0.043	1.000	(1.000, 1.000)	0.044	1.000	(1.000, 1.000)	0.077
**UACR/SACR ratio quartile**									
Q1		Ref			Ref			Ref	
Q2	0.959	(0.618, 1.487)	0.849	0.985	(0.624, 1.553)	0.946	0.948	(0.590, 1.522)	0.821
Q3	1.439	(1.016, 2.038)	0.041	1.458	(1.042, 2.042)	0.029	1.298	(0.904, 1.864)	0.153
Q4	2.645	(1.845, 3.794)	< 0.001	2.766	(1.945, 3.932)	< 0.001	2.382	(1.615, 3.512)	< 0.001
*p* for trend		< 0.001			< 0.001			< 0.001	

*Note:* Model 1 unadjusted. Model 2 adjusted for sex, age, race, education and poverty income ratio. Model 3 was adjusted for variables including sex, age, race, education, poverty income ratio, white blood cell count, platelet count, red cell distribution width, glycated haemoglobin and a history of hypertension or coronary heart disease.

Abbreviations: CI, confidence interval; DR, diabetic retinopathy; OR, odds ratio; UACR/SACR ratio, urinary serum albumin‐to‐creatinine ratio.

### 3.5 Sensitivity and Specificity Analysis

**FIGURE 5:**
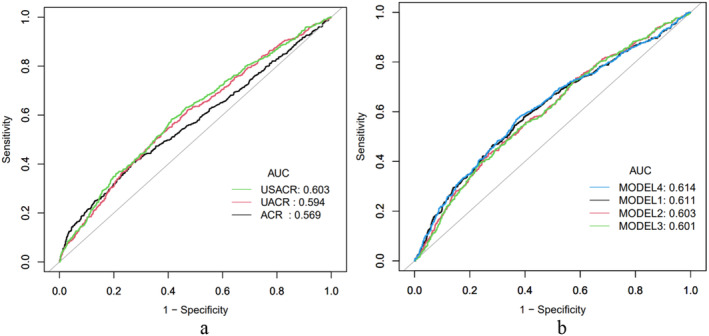
ROC curves for individual biomarkers ROC curves for combined predictive models in predicting DR with HbA1c. AUC, area under the curve; DR, diabetic retinopathy; HbA1c, glycated haemoglobin; MODEL1, ACR + HbA1c; MODEL2, UACR + HbA1c; MODEL3, USACR + HbA1c; MODEL4, ACR + UACR + USACR + HbA1c; ROC, receiver operating characteristic; SACR, serum albumin‐to‐creatinine ratio; UACR, urine albumin‐to‐creatinine ratio; USACR, UACR‐to‐ACR ratio.

We performed receiver operating characteristic (ROC) curve analyses to assess the predictive efficacy of the biomarkers. The ROC curves (Figure 5a,b) are crucial for determining the sensitivity and specificity of SACR, UACR and the UACR/SACR ratio as prognostic diagnostic tools for DR.

Figure 5a illustrates the ROC curves for SACR, UACR and their ratio in predicting DR occurrence. The discriminatory ability of each biomarker to predict DR is measured by the area under the curve (AUC): SACR has an AUC of 0.569, UACR an AUC of 0.594 and the UACR/SACR ratio an AUC of 0.603, reflecting their respective sensitivities and specificities. These values suggest that all three biomarkers have significant predictive value, with the UACR/SACR ratio showing the highest AUC, suggesting it as the most effective predictor among the three. Figure 5b displays the ROC curves for four predictive models incorporating SACR, UACR, UACR/SACR ratio and HbA1c. Model 1, Model 2 and Model 3 each include one of the biomarkers (SACR, UACR and UACR/SACR ratio) along with HbA1c, while Model 4 combines all three biomarkers with HbA1c. The AUC values for Model 1, Model 2, Model 3 and Model 4 were 0.611, 0.603, 0.601 and 0.614, respectively. Model 4, which includes all three biomarkers combined with HbA1c, exhibited the highest AUC, indicating the most robust predictive ability for DR.

### 3.6 Subgroup Analysis

**FIGURE 6:**
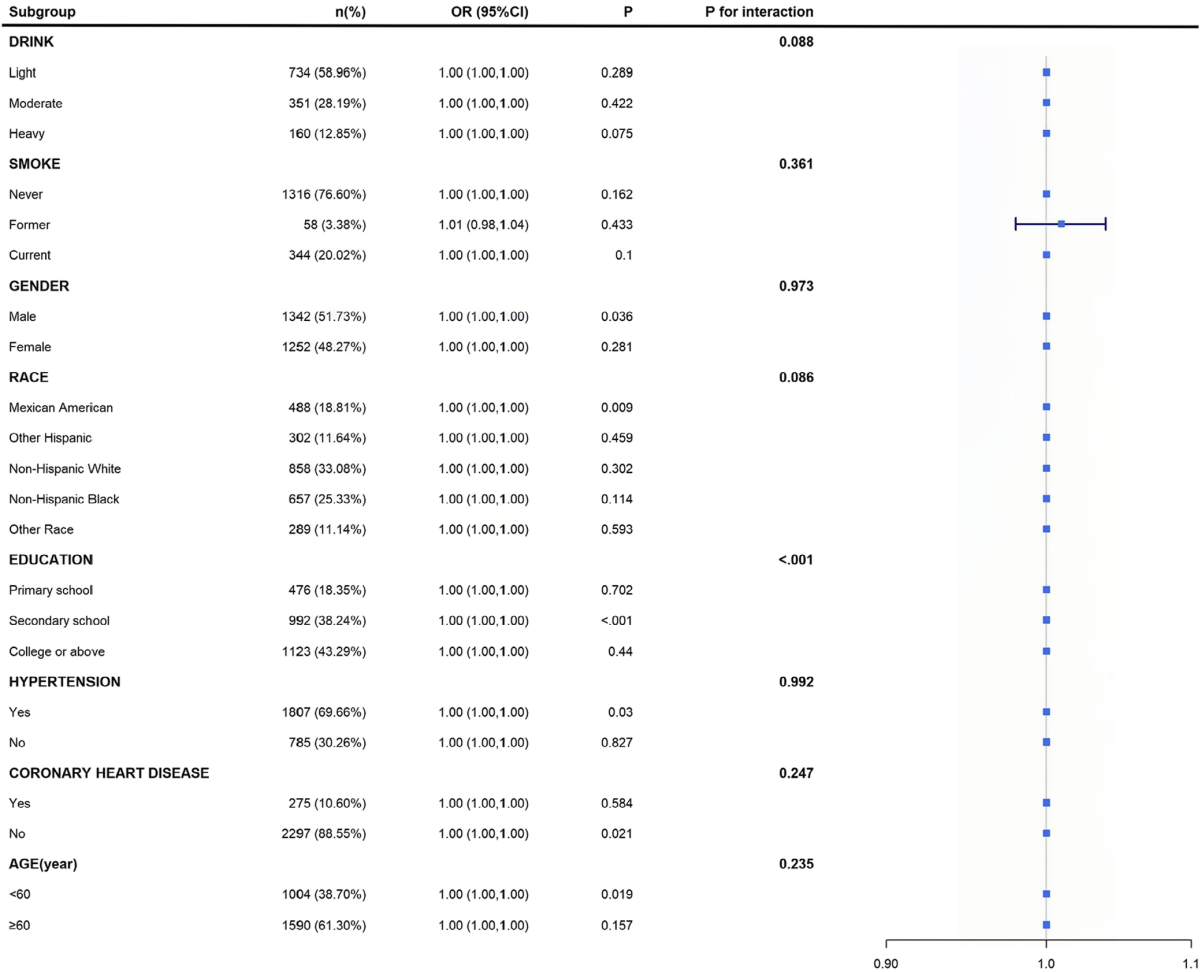
Forest plot of subgroup analyses for the association between urine albumin‐to‐creatinine ratio (UACR)/serum albumin‐to‐creatinine ratio (SACR) and diabetic retinopathy.

Subgroup analyses revealed several notable patterns in the association between UACR/SACR ratio and DR. First, a stronger association was observed in participants aged < 60 years (*p* < 0.05), particularly among those with hypertension. Second, male participants showed a more pronounced association compared to female participants. Third, Mexican Americans demonstrated a stronger relationship between an elevated UACR/SACR ratio and DR compared to other racial/ethnic groups. Interestingly, lifestyle factors, including smoking and alcohol consumption, did not significantly modify the association between UACR/SACR ratio and DR (Figure 6).

## 4 Discussion

This study provides several novel findings regarding the relationships between renal function markers and DR in diabetic patients. First, we found an inverse association between SACR and DR risk, while UACR showed a positive correlation. Second, the UACR/SACR Ratio demonstrated superior predictive capability compared to individual markers. Third, a combined model incorporating SACR, UACR, UACR/SACR ratio and HbA1c showed the highest predictive accuracy (AUC = 0.614), suggesting the complex nature of DR pathogenesis. our subgroup analyses revealed several important findings regarding the association between UACR/SACR ratio and DR across different populations. Notably, we observed a more pronounced association in participants aged < 60 years (*p* < 0.05), suggesting that younger diabetic patients might be particularly susceptible to DR when showing an elevated UACR/SACR ratio. Additionally, patients with hypertension demonstrated a stronger association between UACR/SACR ratio and DR, indicating a potential synergistic effect between blood pressure and microvascular complications. These findings provide new insights into the complex relationship between renal function markers and DR in specific patient subgroups.

The protective role of elevated SACR levels can be explained through multiple molecular and cellular mechanisms. At the molecular level, albumin functions as a potent antioxidant through (1) direct ROS scavenging, (2) pro‐oxidant molecule chelation and (3) cellular redox homeostasis maintenance [17, 18, 19]. These antioxidant properties are particularly crucial in DR pathogenesis, where oxidative stress drives microvascular damage [20]. At the cellular level, albumin exhibits protective effects through anti‐inflammatory and endothelial‐stabilising mechanisms [21], modulating inflammatory mediators and maintaining vascular integrity [22, 23].

The UACR/SACR ratio represents a novel composite marker reflecting the complex interplay between protective and pathogenic factors in DR development. The biological plausibility of the UACR/SACR ratio as a DR predictor is supported by several underlying mechanisms. First, endothelial dysfunction plays a crucial role, reflecting the balance between systemic endothelial protection and damage. This dysfunction manifests through parallel progression in both glomerular and retinal vessels, ultimately affecting microvascular permeability [24]. Second, inflammatory pathways contribute significantly to DR development through multiple mechanisms. These include enhanced expression of adhesion molecules, increased production of pro‐inflammatory cytokines and activation of inflammatory cells in the retinal microvasculature. The inflammatory response creates a cascade effect that further promotes vascular damage and disease progression [25, 26, 27, 28]. Third, oxidative stress represents another critical mechanism linking the UACR/SACR ratio to DR risk. This relationship is characterised by the balance between antioxidant capacity and oxidative damage. When this balance is disrupted, compromised antioxidant defence mechanisms lead to increased oxidative stress, contributing to microvascular complications in both renal and retinal tissues [29, 30, 31].

The positive correlation between UACR and DR risk confirms previous findings [11, 12] while extending them through the incorporation of SACR and the UACR/SACR ratio. The observed dose–response relationship indicates a biological gradient between renal function and DR, suggesting a progressive risk increase with worsening renal function [32].

The subgroup analysis findings have important clinical implications for targeted screening and intervention strategies. The stronger association observed in younger patients (< 60 years) suggests that this age group might benefit from more intensive monitoring of the UACR/SACR ratio for early DR risk assessment. Similarly, the enhanced association in hypertensive patients indicates that this population might require more careful attention to both blood pressure control and UACR/SACR ratio monitoring. These findings support the development of age‐ and comorbidity‐specific screening protocols in diabetes care.

Our study demonstrates several notable strengths that enhance the reliability and generalizability of our findings. The use of a diverse NHANES cohort significantly improves the external validity of our results across different populations. Furthermore, our thorough control for potential confounding variables strengthens the internal validity of the study. The innovative examination of combined biomarkers represents a significant advancement in DR research methodology [33]. Additionally, our comprehensive consideration of inflammatory pathways provides valuable insights into the underlying disease mechanisms.

Despite these strengths, several limitations warrant careful consideration in interpreting our findings. First, the cross‐sectional design inherently limits our ability to establish causal relationships between UACR/SACR ratio and DR development, particularly in determining whether the stronger associations observed in younger and hypertensive patients persist over time. Second, although we made extensive efforts to control for confounding factors, residual confounding may persist, especially regarding systemic inflammation and other microvascular complications. Third, the relatively small sample sizes in certain subgroups, particularly when stratified by age and comorbidities, might limit the statistical power to detect significant associations. Fourth, the limited availability of certain inflammatory markers in our dataset restricted our ability to explore additional pathophysiological pathways that might explain the observed age‐ and hypertension‐related differences. Finally, the lack of validation in independent cohorts necessitates further confirmatory studies to establish the generalizability of our findings, especially regarding the subgroup‐specific associations. The clinical implications of our findings extend beyond conventional risk prediction. The UACR/SACR ratio demonstrates superior predictive capability, suggesting its potential utility in clinical practice through multiple pathways. This ratio enhances early detection capabilities and enables improved risk stratification among diabetic patients. Furthermore, it facilitates more targeted intervention strategies based on individual risk profiles. Importantly, the ratio can be readily integrated with existing diabetes care protocols, making it a practical tool for clinical implementation.

Future research directions should address several key areas to advance our understanding and clinical application of these findings. First, prospective validation studies in diverse populations are essential to confirm the predictive value of the UACR/SACR ratio, particularly focusing on age‐specific associations and their interaction with hypertension. Second, mechanistic studies should explore the age‐related differences in microvascular pathology and inflammatory responses, incorporating comprehensive inflammatory marker panels to provide deeper insights into the stronger relationship observed in younger patients. Third, investigation of additional microvascular complications could reveal broader applications of these biomarkers and their potential role in early disease detection. Fourth, the development of standardised implementation protocols, including specific monitoring strategies for high‐risk populations such as younger patients with hypertension, would facilitate consistent clinical application. Finally, cost‐effectiveness analyses across different healthcare settings would inform practical implementation decisions.

The clinical implications of our findings extend beyond conventional risk prediction. The identification of UACR/SACR Ratio as a potential predictor of DR, particularly its enhanced predictive value in specific subgroups, opens new avenues for personalised medicine in diabetes care. While validation in independent cohorts is needed before widespread clinical implementation, our findings provide a strong foundation for risk stratification and targeted intervention strategies, potentially improving the early detection and management of DR in high‐risk populations.

## 5 Conclusion

In conclusion, our study demonstrates that the combined predictive model incorporating SACR, UACR, UACR/SACR ratio and HbA1c (AUC = 0.614) significantly outperforms individual biomarkers in DR risk assessment. This superior predictive capability reflects the complex pathogenesis of DR and underscores the value of a comprehensive approach to risk evaluation. Furthermore, our subgroup analyses revealed important demographic and clinical modifiers of this relationship, with particularly strong associations observed in patients aged < 60 years and those with hypertension. These age‐ and comorbidity‐specific findings suggest the need for targeted screening approaches in these high‐risk populations.

The novel UACR/SACR ratio, when integrated with traditional markers, not only enhances the model's predictive accuracy but also demonstrates varying predictive strength across different patient subgroups. This heterogeneity in association strength supports the potential for more personalised risk assessment strategies, particularly for younger patients and those with concurrent hypertension. These findings collectively contribute to our understanding of DR risk stratification and suggest the possibility of age‐ and comorbidity‐specific screening protocols.

Prospective studies are needed to validate these findings, particularly the subgroup‐specific associations, and to establish their clinical implementation. Future research should focus on understanding the mechanisms underlying the age‐specific differences in association strength and the interaction between hypertension and the UACR/SACR ratio in DR development. Such validation and mechanistic studies will ultimately lead to improved screening protocols and personalised management strategies for diabetic patients at risk of DR, particularly those in high‐risk subgroups.

## Author Contributions

**Han Dai:** formal analysis, data curation, visualization and writing – original draft. **Ling Liu:** conceptualization, methodology, investigation and writing – review and editing. **Weiwei Xu:** conceptualization, methodology, supervision, project administration and writing – review and editing. All authors have read and agreed to the published version of the manuscript.

## Conflicts of Interest

The authors declare no conflicts of interest.

## Data Availability

The datasets generated and analysed in this study are available on the National Health and Nutrition Examination Survey (NHANES) website at https://www.cdc.gov/nchs/nhanes/index.htm.
